# Socio-economic, demographic, and environmental characteristics of child drowning on Sandwip Island, Bangladesh

**DOI:** 10.3389/fpubh.2026.1844029

**Published:** 2026-08-12

**Authors:** Edris Alam

**Affiliations:** 1 Faculty of Resilience, Rabdan Academy, Abu Dhabi, United Arab Emirates; 2 Department of Geography and Environmental Studies, University of Chittagong, Chittagong, Bangladesh; 3 Disaster and Development Organization (DADO), Chattogram, Bangladesh

**Keywords:** Bangladesh, child, death, drowning, poverty, safety, socio-demographic

## Abstract

**Objective:**

This study aims to identify the perceived risk factors associated with child drowning deaths on Sandwip Island⎯ a region of high drowning deaths over generations.

**Methods:**

This study was conducted using a purposive sampling survey to identify fatal and nonfatal child (1–17 years of age) drowning among a total of 13,857 households in two unions, namely Sarikait and Maitbanga in Sandwip Island, Bangladesh from 2018–2023. Face-to-face interviews were conducted among the households who experienced drowning on the Island. We used a combination of methods for data collection, incorporating both quantitative analyses to assess socio-economic, demographic, and environmental factors, and qualitative analysis to investigate situational factors related to drownings in the region. The annual incidence rate of fatal drowning was estimated by determining the number of events occurring per 100,000 persons.

**Results:**

By visiting 13,857 households, a total of 205 households were identified that faced drowning incidents between 2018 and 2023. Out of 205 child drowning incidents in the 2 unions studied, 117 (57.07%) were fatal and 88 (42.93%) were non-fatal. In 2023, the number of drowning incidents was 23. Annual fatal drowning rate on the Island was 20.85/100,000 in 2023. Out of 117 fatal drowning deaths, 69.2% occurred in children between the ages of 1–4 years old. Prior to drowning incidents, approximately 88% of children were without active adult supervision. The average age for 117 drowning deaths is 3.2 years. Of the child drowning deaths, 62 (53%) were male and 55 (47%) were female. The gender proportion of 88 non-fatal drownings were 60% male and 40% female. Regarding seasonality of incidences, the occurrence in summer (March to May), monsoon (June to October) and winter (November to February) were 31.69, 39.04 and 29.27%, respectively. Of drowning deaths, 96.58% occurred in ponds, 2.56% occurred in ditches and 0.85% occurred in cow’s fodder bucket. From these 117 fatal drownings, approximately 98% of children did not know how to swim prior to the incident. The average age at which children first learned to swim was 7 years.

**Conclusion:**

The findings indicate that inadequate active adult supervision at the time of the incident, the high density of ponds, and locational factors—particularly the proximity of household compounds to nearby water bodies—collectively contribute to elevated drowning risk. Identifying socio-demographic and reported contributing risk factors associated with child drowning can inform the development of targeted prevention strategies, including interventions that promote earlier acquisition of swimming skills to reduce incidence among younger age groups.

## 1 Introduction

Drowning is defined as “the process of experiencing respiratory impairment from submersion or immersion in liquid” (1). It remains a major but relatively neglected global public health problem, particularly among children and young adults. In 2021, approximately 300,000 people died from drowning worldwide, making it one of the leading causes of unintentional injury mortality (2). The burden of drowning is disproportionately concentrated in low- and middle-income countries (LMICs), which account for nearly 90% of global drowning deaths (3). The highest mortality rates occur in the WHO South-East Asia and Western Pacific regions, where drowning rates substantially exceed those reported in many high-income countries (2). Socio-economic disadvantages, limited supervision, unsafe water environments, inadequate infrastructure, and restricted access to prevention measures contribute to the elevated drowning burden in LMIC settings (4–6). Across these contexts, children living in rural and resource-constrained communities are particularly vulnerable to drowning exposure around household and community water bodies.

Child drowning represents a critical public health issue in Bangladesh, where drowning is one of the leading causes of death among children aged 1–17 years and exceeds deaths from pneumonia, malnutrition, and diarrheal diseases combined (7). The highest drowning risk occurs among children under 5 years of age, especially males and those from socio-economically disadvantaged households (8, 9). Previous studies in Bangladesh have identified several contributing factors, including inadequate child supervision during working hours, proximity of open water bodies to households, low maternal education, seasonal flooding, and poverty (8, 10). Most drowning incidents involving young children occur during daytime hours and within close distance of the home, frequently in ponds, ditches, buckets, or other domestic water hazards (9, 11, 12). Bangladesh’s low-lying deltaic landscape, recurrent flooding, and widespread household pond systems further increase children’s exposure to water-related risks.

Despite growing evidence on childhood drowning in Bangladesh, limited research has examined the socio-demographic, geographic, and environmental determinants of drowning in island settings. Sandwip Island represents a distinctive context because of its geographic isolation, rural settlement patterns, dependence on pond-based water systems, and environmental vulnerability. For generations, drowning in ponds and nearby water bodies has remained a major cause of child mortality on the island. Improved understanding of the contextual drivers of drowning is essential for developing effective and locally appropriate prevention strategies. Therefore, this study aims to identify the socio-demographic, geographic, and environmental factors associated with child drowning on Sandwip Island, Bangladesh, and to provide evidence that may support targeted drowning prevention interventions in similar rural and coastal settings.

## 2 Research methodology

To examine perceived multidimensional risk factors underlying child drowning, we implemented a mixed-methods approach that integrates quantitative spatial-demographic assessments with qualitative contextual analysis (13). Specifically, closed-ended survey questions captured socio-economic, demographic, and environmental hazards, while open-ended interviews, case studies, and field observations captured localized, situational dynamics. Although each method has its limitations, mixed approaches yield more comprehensive and reliable results (14, 15).

A survey on drowning incidents was conducted on Sandwip Island, located off the Bay of Bengal. Sandwip Island is a sub-district in the Chittagong district, comprising 15 unions. The island’s total population is 327,553, with the number of households to be determined. Each union, the lowest level of local government, is divided into nine wards. For the field survey, the Sarikait and Maitganga unions were selected based on the availability of volunteer enumerators, the willingness of respondents to participate in the survey, and the authors’ prior knowledge of drowning incidents in the region. In 2022, Sarikait Union had a population of 34,315 and 7,395 households, while Maitbanga Union had a population of 24,702 and 6,462 households. The total number of populations in both unions is 59,017. We began by establishing drowning data from these two unions through visits to 13,857 households. The survey collected information on age and sex characteristics, timing and seasonality, and reported environmental factors for fatal and non-fatal drownings in the targeted households between 2018 and 2023. For the purposes of this study, an interrupted drowning process resulting in survival at any point was classified as a non-fatal drowning, whereas a drowning incident resulting in death at any point was classified as a fatal drowning. Crucially, any submersion or immersion incident that did not result in respiratory impairment was classified as a water rescue rather than a drowning. Identifying socio-demographic and other perceived risk factors associated with child drowning will aid in developing effective prevention strategies and interventions for these regions and beyond.

### 2.1 Collecting drowning occurrences between 2018–2023

Over a three-month period (January–March 2024), we conducted a survey to estimate drowning incidents and identify socio-demographic characteristics and other perceived risk factors, such as location, time, and circumstances of child drownings on the island. To comprehensively document child drownings, we developed a database and interview format based on the child drowning risk estimation index by Borse et al. (16), water incidence index by Hills et al. (17) and the WHO guidelines for community surveys on injuries and violence (18). Data were collected through face-to-face interviews using a pretested format that included structured, semi-structured, and open-ended questions. The interview tool had two sections: the first focused on socio-economic and demographic characteristics (e.g., age and sex of the drowning child, mother’s age, parents’ professions, and household income), and the second on drowning specifics (e.g., location, time, activities before drowning, water depth, distance from home, accompanying person’s activity, swimming ability, and drowning situations).

Eighteen data collectors, one for each ward in a union, and two supervisors, one for each union, were employed for the data collection process. All data collectors and supervisors were trained on the study objectives, interview questions, and ethical protocols. The survey focused exclusively on households that had experienced child drowning incidents. A household refers to a person or a group of people, related or unrelated, who usually reside in the same dwelling unit and share meals prepared from a common kitchen. Investigators conducted a house-to-house screening across the designated wards to determine eligibility. Accordingly, all households within the two unions were visited to identify cases of child drowning that occurred between 2018 and 2023. During the visit, respondents were asked whether a child in the household had experienced a drowning event. Only those households reporting a fatal or non-fatal drowning incident during this period were included for interview. If the drowning site was near the household, data collectors measured the distance visually. Repeat visits were made if respondents were unavailable during the first visit. Thus, a total of 205 households were identified that faced drowning incidents during study period. Interviews were conducted in Bengali by native speakers, and responses were translated and transcribed into English by a certified translator.

### 2.2 Ethical issues

Ethical safeguards included trauma-informed interviewing by trained researchers, obtaining informed consent, allowing participants to pause or withdraw at any time, minimizing intrusive questioning, and providing referrals to appropriate psychosocial support services where needed. Personal identifiers were removed, and pseudonyms were used to maintain confidentiality. The study was approved by the Research and Ethics Committee of Rabdan Academy (RAREC00040) and the Ethics Committee of the University of Chittagong, Chittagong 4,331, Bangladesh (CUNI# 0179). Clinical trial number: not applicable.

### 2.3 Data analysis

Data from the survey were used to estimate drownings in between 2018 and 2023, categorized by sex, age group, location, seasonality, and time of drowning. Participants were also categorized by socio-demographic characteristics. We used MS Excel to calculate the male-to-female respondent ratio, income levels, the number of child drowning incidents, swimming ability prior to drowning and the age-sex distribution of fatal and nonfatal drownings. Incidence rates for child drowning were used to quantify the frequency of child drowning events. Following the standard public health injury surveillance practice [1], age-specific denominators were used to prevent the “denominator dilution effect” of using the total population. The total number of drownings reported divided into fatal drowning deaths and non-fatal drowning survival events. Formula of Incident Rate is (Number of Incidents/Child Population 1–17) × 100,000. *Denominator*: The number of children aged 1–17 years who reside in the study unions who are at risk (*n* = 19,180). This baseline denominator profile was extracted from the age-stratified demographic data of the National Population and Housing Census 2022. Multiplier: Results are expressed as events per 100,000 child-years of exposure. Quantitative findings were presented as hierarchical frequency tallies ordered according to their total counts and relative ranking (Tables 1–6).

**Table 1:** Demographic and socioeconomic characteristics of the respondents.

Items/characteristics	Frequency	Percentage
Gender		
Male	106	51.71
Female	99	48.29
Family type		
Nuclear	163	79.51
Extended	42	20.49
Age of the respondents		
18–24	24	11.71
25–34	69	33.66
35–44	62	30.24
45–54	31	15.12
54–64	18	8.78
65+	1	0.49
Number of family members		
1–4	47	22.93
5–7	158	77.07
Education level		
Illiterate	32	15.61
Primary	58	28.29
Secondary	103	50.25
Higher education	12	5.85
Major occupations		
Housewives	97	47.32
Small trading	43	20.97
Day laborer	14	6.83
Unemployed	14	6.83
Agriculture	13	6.35
Service	10	4.88
Remittance earner	7	3.41
Fisher	7	3.41
Monthly income (BDT)		
0–5,000	77	37.56
5,001–10,000	36	17.56
10,001–15,000	35	17.07
15,001–20,000	26	12.68
20,001–25,000	18	8.78
25,001–30,000	6	2.93
35,001–40,000	4	1.95
More than 40,000	3	1.47

**Table 2:** Distribution of drowning incidents by wards and unions between 2018 and 2023.

Ward number	Sarikait union		Maitbanga union	
	Frequency	Percentage	Frequency	Percentage
1	5	4.24	4	4.60
2	18	15.25	11	12.64
3	27	22.88	7	8.05
4	6	5.08	6	6.90
5	12	10.17	13	14.94
6	8	6.78	7	8.05
7	14	11.86	8	9.20
8	12	10.17	15	17.24
9	16	13.56	16	18.39
Total	118	100	87	100

**Table 3:** Drowning per annum in the study region between 2018 and 2023 (*n* = 205).

Year	Sarikait union				Maitbanga union				Total	
	Fatal		Non-fatal		Fatal		Non-fatal		Frequency	Percentage
	Frequency	%	Frequency	%	Frequency	%	Frequency	%		
2023	2	3.08	9	16.98	2	3.85	10	28.57	23	11.22
2022	4	6.15	7	13.21	3	5.77	2	5.71	16	7.80
2021	4	6.15	8	15.09	2	3.85	3	8.57	17	8.29
2020	5	7.69	5	9.43	2	3.85	4	11.43	16	7.80
Before 2020	50	76.92	24	45.28	43	82.69	16	45.71	133	64.88
Total	65	100	53	100	52	100	35	100	205	100

**Table 4:** The reported activities of children prior to drowning.

Child doing prior to drowning	Frequency	Percentage
1. Went to play outside whilst mother was busy with household chores	134	65.85
2. Playing in the courtyards alone	16	7.80
3. Under the mothers’ supervision	16	7.32
4. Kept inside the house without barriers	10	4.88
5. Went to bathe in the pond alone	9	4.39
6. Playing with friends close to the pond	8	3.90
7. Water sports with peers in the ponds	7	3.42
8. Playing with siblings	4	1.95
9. Under grandparent’s supervision	1	0.49
Total	205	100

**Table 5:** The place of fatal and non-fatal drownings between 2018 and 2023.

Place of drowning	Fatal		Non-fatal	
	Frequency	Percentage	Frequency	Percentage
Pond	113	96.58	87	98.86
Ditch	3	2.56	1	1.14
Cow’s fodder bucket	1	0.85	—	—
Total	117	100	88	100

**Table 6:** The reported social, economic, or geographic factors of drowning.

Reported social, economic, or geographic causes of drowning	Frequency	Percentage
Pond very close to the settlement	169	82.44
Poverty	16	7.80
Impoverished house	9	4.39
Not available	9	4.39
Broken and damaged culverts	2	0.98
Total	205	100

Qualitative data were analysed using a systematic and iterative approach. Responses to semi-structured and open-ended questions were initially organized according to conceptually informed themes and subsequently examined using both manual techniques and computer-assisted tools (MS Word and Excel). Participant narratives were transcribed and translated into standard Bengali, after which emerging patterns were identified through content analysis (35). Sub-themes were consolidated into broader categories that reflected the study’s objectives. For example, we identified themes from similar narratives, including children’s activities before drowning, the drowning locations, financial difficulties leading to inadequate supervision, and the absence of barriers around water bodies. Qualitative responses such as “busy with household chores,” “child playing in the courtyard,” “had not heard any noise for a while,” and “child playing with peers” were identified from open-ended questions. The results were interpreted based on relevant theoretical and development literature. To strengthen the validity of these categories, triangulation was undertaken with complementary sources, including survey data, field notes, and direct observations, while recognizing the reliance on participants’ retrospective accounts. Case studies were developed from notes taken during the field surveys. The final interpretations were developed in reference to relevant theoretical frameworks and contemporary development literature. Qualitative findings were provided alongside quantitative data to offer a deeper understanding of the responses, aligned with the study’s questions.

## 3 Results

### 3.1 Socio-demographic characteristics of the participants

Out of the 205 individuals surveyed, 51.71% were male and 48.29% were female (see Table 1). Regarding family structure, 79.51% belonged to nuclear families (comprising parents and children), while 20.49% were part of extended families (including grandparents, parents, children, or siblings living with their spouses and children). The average family size was 6.1. The average monthly income was USD 98 (with USD 1 equivalent to BDT 122.79 as of May 2026). About 56% of the participants had a monthly income of less than USD 83. In total, 15.61% of the respondents were illiterate. Educational attainment among the participants was as follows: 28.29% had primary education, 50.25% had secondary education, and 5.85% higher education. In total, 47.32% of respondents were housewives. Other occupations included small traders (20.97%), day laborers (6.83%), unemployed (6.83%), agricultural workers (6.35%), service sector workers (4.88%), remittance earners (3.41%), and fishers (3.41%). In total, 72.2% of the homes were constructed from corrugated iron sheets. Of the remaining homes, 18.05% were single-story brick houses, 8.29% were semi-brick houses, and 1.46% were made of bamboo.

### 3.2 Geographic context of the respondents

By visiting a total of 13,857 households, we identified 205 drowning incidents across the Sarikait and Maitbanga unions. Of these incidents, 57.56% occurred in Sarikait Union and 42.44% in Maitbanga Union. The distribution of drowning incidents by ward is presented in Table 2. In Sarikait Union, ward # 3 had the highest number of drownings (22.88%), while ward # 1 had the lowest (4.24%). In Maitbanga Union, the highest incidence of drownings (18.39%) was in ward # 9, with ward # 1 experiencing the fewest (4.60%).

### 3.3 Age and sex of child drowning

**Figure 1:**
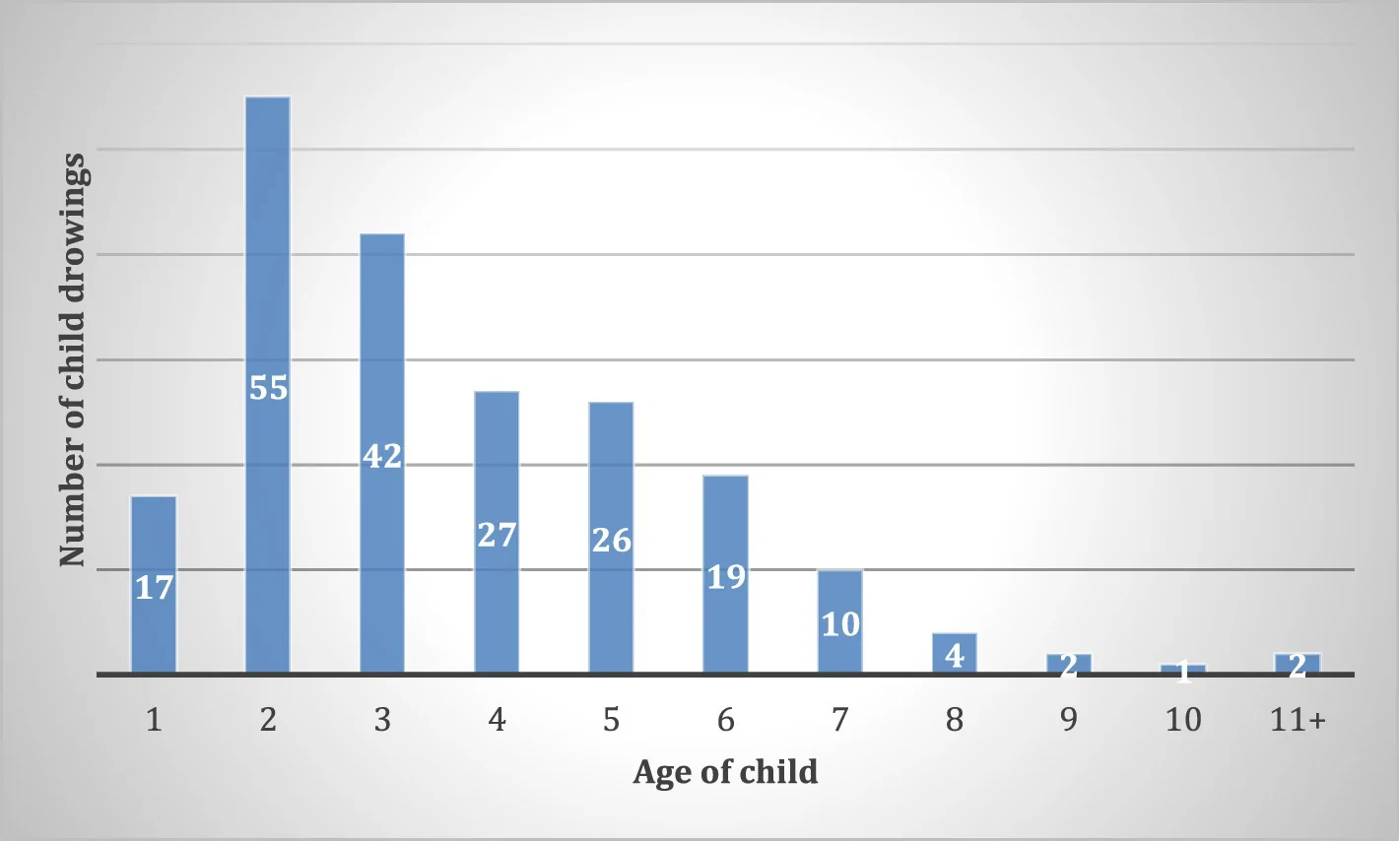
Age distribution of child drowning cases occurring between 2018 and 2023 (*n* = 205).

Among the 205 child drowning incidents in the two unions studied, 117 (57.07%) were fatal and 88 (42.93%) were nonfatal. In 2023 alone, there were 23 (11.22%) drowning incidents (see Table 3). Annual fatal drowning rate on the Island was 20.85/100,000 in 2023. Of the 117 fatal drownings, 69.20% involved children aged 0–4 years (Figure 1), with the average age of these fatalities being 3.2 years. Among the fatal cases, 62 (53%) were boys and 55 (47%) were girls. For the 88 non-fatal drownings, 60% were boys and 40% were girls. Non-fatal drownings did not occur in children older than 9 years, and fatal drowning decreased after the age of seven. Over 95% of drownings occurred in children under 8 years (Figure 1).

### 3.4 Swimming ability prior to drowning

Of the 117 fatal drownings, nearly 98% of the children did not know how to swim. Among the 88 non-fatal drowning incidents, caregivers reported that none of the children knew how to swim prior to the incident. The average swimming learning age is 7 years. From the 205 respondents, 32% reported limited opportunities for teaching children to swim, mainly due to pollution in pond water. Discussion with participants suggests that the region lacks designated swimming areas, relying solely on traditional ponds for bathing and swimming.

### 3.5 Time and seasonality of drowning

**Figure 2:**
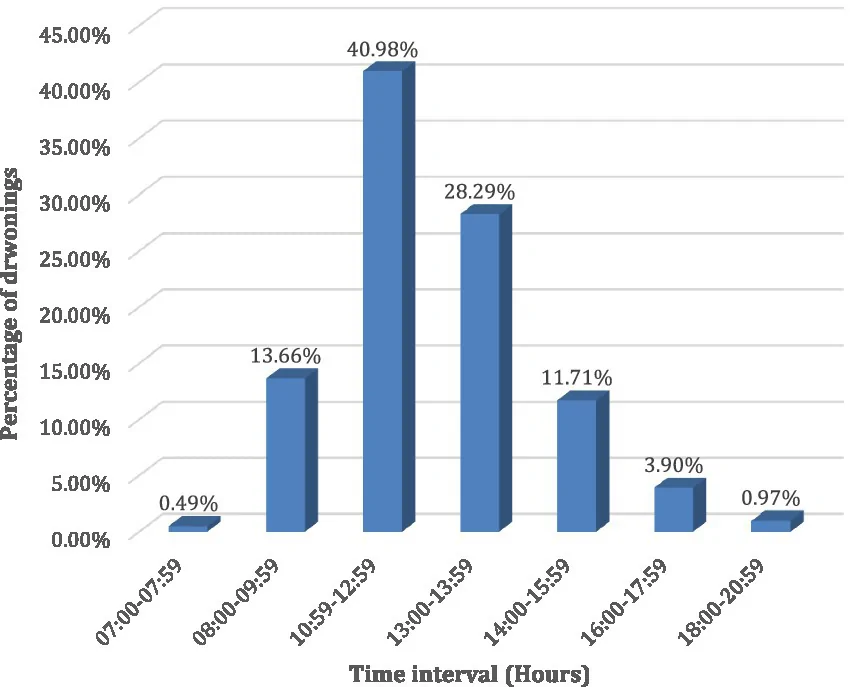
The distribution of child drowning incidents by time intervals (hours). Most drownings occurred between 8 a.m. and 2 p.m.

The findings indicate a notable pattern in the timing and seasonality of drownings in the region. Of the 205 drownings, 83.42% occurred between 8 a.m. and 2 p.m., a period when households are typically engaged in chores (see Figure 2). The highest peak period for drownings was between 10 a.m. and 1 p.m., accounting for 40.98% of incidents. Seasonally, drownings occurred 31.69% in summer (March to May), 39.04% during the monsoon season (June to October), and 29.27% in winter (November to February).

### 3.6 The activities of children prior to drowning

Prior to drowning, only 7.80% of children were under parental supervision, while the remainder engaged in various activities. These activities were classified into nine distinct categories. (see Table 4). The top five activities were: (1) going outside to play while the mother was occupied with household chores; (2) playing alone in the courtyard; (3) being under the mother’s supervision; (4) staying inside the house; and (5) going to bathe in the pond alone.

A parent residing in ward number 7 of Maitbanga union account:

*Our thirteen-month-old child was playing in the yard with the six-year-old older brother. I was busy with housework. When I finished, I saw that the child was not there. When I asked the older brother, he said that someone from the house had taken her to a neighbor’s house. I went to that house to bring back the child, but I was told that the child did not go there. After that we went to surrounding areas and found her floating upside down in the water. The child recovered from the pond was already deceased*.

### 3.7 Locational attributes of child drowning

**Figure 3:**
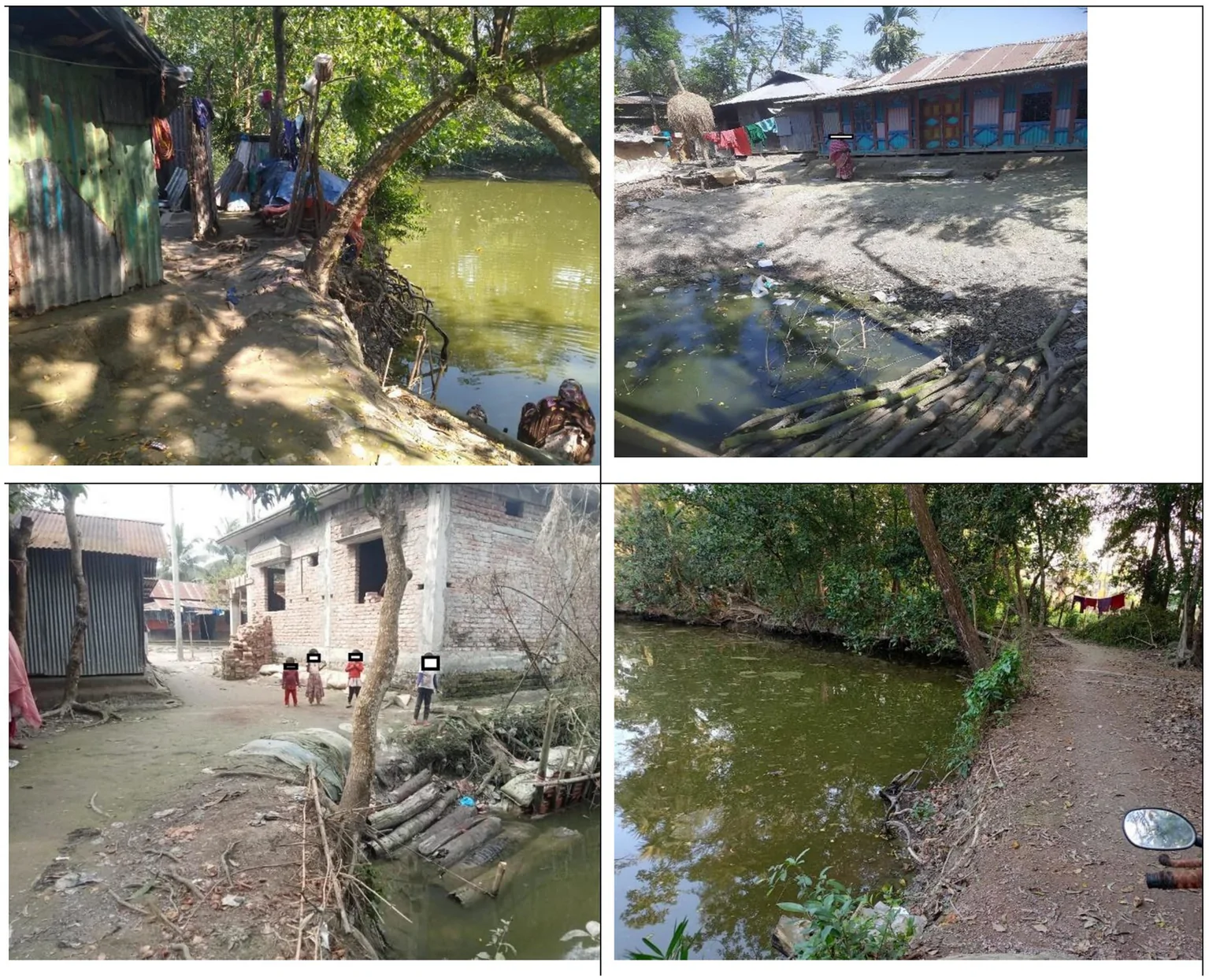
Location of ponds and houses on Sandwip Island. The absence of barriers between ponds and nearby houses poses a significant risk for child drowning.

To protect houses from rainwater and frequent tidal surge from the adjacent Bay of Bengal, the island people dig ponds to raise the plinth of the houses. We noted that the owner of a single house also digs ponds to raise the courtyard and plinth of the house. On some occasions, three to four ponds are located within 50–100 meters of residential houses. These ponds are generally used for bathing and washing and meet nutritional needs of households from raised fish in the ponds (Figure 3). These ponds, usually over seven feet deep and located 20 to 30 meters from residences, increase the drowning risk for children. Among the 117 fatal drowning incidents, 96.58% occurred in ponds, 2.56% in ditches, and 0.85% in cow’s fodder buckets (see Table 5). For the 88 non-fatal drownings, 98.86% happened in ponds and 1.14% in ditches. The average water depth in the ponds where 117 fatal drownings occurred was 6.18 feet. Across all 205 drownings, the average distance from the incident location to the house was 25.6 meters. Through field visits and observations of drowning locations on the island, it was noted that children’s play yards are often situated between the house and adjacent ponds. Parents commonly leave children playing in the yard while they attend household chores.

To better explain proximity between houses and ponds causing child drowning (Figure 3), it could be understood from a participant’s account. A mother who is 23 years old stated:

We are deeply heartbroken over the loss of our beloved 2-year-old son. It happened in November 2023. We were a new family, just my husband, our only son, and myself. My husband typically leaves early in the morning for work and returns home for a 30-min break around noon. It was a typical day for me, leaving our son in the yard to play while I managed the household chores. I remember he was with me in the yard as I prepared firewood for cooking. Once the wood was ready, I went inside to cook lunch, knowing my husband would soon return. The kitchen was hot and smoky from the wood fuel, so I could not allow our son inside. Without a window in the kitchen, I could not see him anyway and had to trust that he was playing safely in the nearby yard. After some time, my heart suddenly filled with dread—where was my child? I rushed out of the kitchen and looked around where he usually played. Hearing my cries, our neighbours gathered and began searching the area extensively. A young neighbor brought a fishing net and cast it into a nearby pond. Feeling the net was heavy, he jumped into the water and tragically found our son had drowned. The pond was just next to our yard, approximately 10 meters from our house. Our hearts ache with this unimaginable loss.

### 3.8 The reported social, economic, or geographic factors of drowning

Participants were asked to identify the underlying social, economic, or geographic determinants of drowning within the region, independent of caregiver supervision or the child’s immediate activities prior to the incident. The participants mentioned four reasons which are presented in hierarchical order: (1) pond very close to the settlement (82.44%); (2) poverty (7.80); (3) impoverished house (4.39%); (4) broken and damaged culverts (0.98%). Approximately 4.39% could not provide any responses for this question (Table 6).

Out of 117 drowning deaths on the island, 99% occurred in ponds and ditches, which were without any barriers. This open access to ponds allowed children easy entrance. The ponds where child drownings occurred were mostly owned by a couple of families and needed everyone’s consent and financial contribution for fencing. This challenge can be better understood by one participant in Maitbanga. A father stated:

I do not live alone in the house, nor do I solely own the pond. Therefore, I cannot unilaterally provide consent to install a barrier around it. Regrettably, I also lack the financial means to cover the expenses for the barrier on my own. This is an issue that requires collective action. We need to come together with all the pond owners to discuss and reach a consensus. I am concerned that those without young children may be less inclined to agree or contribute financially toward installing the barrier.

## 4 Discussion

The study identified that among 13,857 households, 205 households experienced child drowning incidents in Sarikait and Maitbanga unions. We observed variation in drowning deaths across wards within the study area. We postulate that this is likely attributable to higher population density and the widespread proximity of ponds to residential settlements. In 2023, the island recorded annual fatal drowning rate of 20.85 per 100,000 of the population. This estimate is close to those reported by Rahman et al. (19), who documented higher drowning rates approximately 16.7 to 20.3/100,000 per year based on a substantially larger sample from 51 union parishads in central Bangladesh in 2013. This study identifies specific risk factors associated with child drowning including children aged 2–3 years (47.87% fatal drowning), the monsoon season, time (8 a.m.-2 p.m., 82.93% of drownings) while parents are engaged with household chores, the presence of a pond or ditch without fencing around the household, inability to swim, and limited child supervision. These findings are consistent with previous studies in other regions in Bangladesh (8, 9, 16, 20) and other studies in India, Vietnam and China (21–23).

While global literature typically demonstrates a pronounced male predominance in drowning fatalities—often double the rate of females (5, 24)—the case findings from Sandwip Island reveal a much narrower margin (53% male vs. 47% female). This departure from established trends can likely be attributed to two intersecting factors: population age and environmental exposure. First, literature suggests that male predominance typically widens in older cohorts due to age-progressive exposure and distinct occupational or recreational behaviours. The absence of a stark gender disparity in this study is likely explained by the cohort’s young mean age (3.2 years), a developmental stage marking a critical transition point where constant supervision often declines. While some literature notes that independent mobility can expand more rapidly for young male children at this stage, the overall cohort youth minimizes early gender-specific behavioral divergence. Second, an overwhelming 96.58% of these fatal drownings occurred in adjacent domestic ponds. This represents a highly unique environmental risk profile compared to broader national data, such as Rahman et al. (25), who found that only 43% of drownings occurred in ponds across mixed rural and urban households. Ultimately, these proximate, domestic hazards present an equitable risk to young children of both genders, emphasizing a universal early-childhood vulnerability that overrides macro-level gender trends and warrants further socio-behavioral investigation.

Over 82% of drowning deaths occurred between 8 a.m. and 2 p.m. These are the periods that a household engages heavily with household chores. Afternoon drownings, particularly between 5–6 p.m., were mostly associated with water sports. Relatively less, approximately 39% drowning deaths occurred in monsoon season, a sharp difference to the other region in Bangladesh. Rahman et al. (19) conducted a survey in seven sub-districts in the middle of the country, demonstrated that approximately 75% drowning occurred between June and October, which is the monsoon season when the level of water through seasonal rainfall and flooding rise to the household level in the region. In contrast to Rahman et al. (19) who reported no child drowning incidents in the winter season, our findings suggest that 29.27% drowning deaths occurred on the island in that same season. This means that a one-size-fits-all strategy may not be effective for the entire country. It also highlights the need to understand drowning patterns across diverse geographical, demographic, and socio-cultural contexts.

Approximately 98% of fatal drowning children did not know how to swim. Among parents who taught their children to swim, the average age at which children acquired swimming skills was 7 years. In comparison, a recent study by Alam et al. (8) reported an average learning age of 8.33 years in northern Bangladesh. The survey results indicate that approximately 25% of children who drowned were between the ages of 5 and 7. This implies that households should be encouraged to teach children about the dangers of water and how to swim from earlier ages. The ability to swim is a basic survival skill that protects against water related incidents (26). The WHO recommends swimming and water safety instruction for school-aged children as a key component of drowning prevention strategies (27). Rahman et al. (5) noted that considering health conditions, Bangladeshi children can start learning to swim from the age of six. However, a review of studies by Olivar and Moreno-Murcia (36) suggested that for children of 10 to 14 years, knowing how to swim did not significantly reduce drowning compared to those who did not know how to swim. Similarly, a recent scoping review on the effectiveness of drowning prevention interventions for individuals under 20 years concluded that the evidence supporting swimming instruction as an effective strategy for children under 6 years does not support generalizable recommendations (28). On the contrary, our study found limited children facing fatal drowning after the age of eight and no nonfatal incidents after the age of nine among those who have already acquired swimming skills. As this study shows, the decline in traditional pond swimming due to pollution and the lack of infrastructure highlights the need to expand designated swimming centers in the region.

The findings have significant policy implications about the need for active child supervision and fencing around pond walkways. Approximately 97% of fatal drowning incidents occurred in ponds, suggesting that fencing ponds located near households and walkways may be a feasible preventive measure. Although the geographical and socio-economic contexts differ, a recent study by Bista and Michaels (29) reports similar findings in the United States, where deaths of young children due to inadequate supervision around unfenced ponds were prevalent, highlighting the need for improved supervision and stricter enforcement of fencing regulations. Such interventions require community consensus and shared financial responsibility. Some other community-based cost-effective measures may include raising awareness among the population about drowning risk and particularly about the importance of not allowing children to play unsupervised. Additional community-based interventions, such as training bystanders in safe rescue practices as recommended by the WHO, may also be implemented on the island as a preventive measure. Along with individual and community-level awareness and capacity building, effective child drowning prevention will require the involvement of both local and central governments in developing appropriate prevention packages that reflect localized contextual factors and include community consultations. Specific interventions, such as swimming lessons for young children, community awareness programs, and construction of barriers around water bodies, should be implemented and evaluated for their effectiveness in reducing drowning incidents.

The earlier pilot project implemented in seven rural sub-districts proved that crèches are both highly effective and cost-efficient as primary strategies to prevent drowning among children under five in Bangladesh (11, 30, 31). As noted earlier, the average age of drowning victims was 3.5 years, a developmental stage marked by increased mobility, curiosity, and risk-taking behavior. These characteristics highlight the need for adequate supervision or appropriate childcare arrangements when primary caregivers are engaged in work or other activities. However, crèches facilities were completely absent in the region. Following its success, this initiative has been expanded and transferred to the Bangladesh government and is now being implemented in select districts by the Bangladesh Shishu Academy.

Sandwip Island is highly exposed to cyclone landfall events and has experienced catastrophic mortality during the major cyclones of 1970 and 1991, each causing approximately 10,000 deaths (32). Over the past two decades, the active implementation of the Cyclone Preparedness Program (CPP), including public awareness efforts, timely evacuation, and other preventive measures, has contributed to a substantial reduction in cyclone-related fatalities (33). Further research is needed to determine how drowning prevention strategies can be integrated into existing disaster risk reduction, climate change adaptation, health programs, and broader development initiatives to ensure effective and cost-effective drowning prevention in this region.

This study has several limitations that need to be considered. Firstly, it relies on participants’ memories of drowning incidents in the past 6 years, which may lead to recall bias. While this recall period may introduce recall bias, with better recall of fatal events than non-fatal ones, structured questionnaires and detailed probing during interviews were used to enhance accuracy. However, such bias cannot be fully eliminated completely in such studies. Due to the absence of systematic drowning data recorded by government departments on the island, we were unable to cross-check our survey results with official local data. Additionally, the findings are specific to two unions in Sandwip Island and may not apply to other regions with different socio-economic and environmental contexts. To improve the generalizability of the findings, future research should cover a wider geographic area. Furthermore, the study uses a cross-sectional design, which restricts the ability to determine causality. Longitudinal studies are necessary to better understand the causal relationships between socio-demographic factors and drowning incidents. As no non-drowning comparison group was included, risk factor estimation and causal inference were not possible. Accordingly, findings are interpreted as descriptive associations among drowning cases rather than determinants of drowning risk. Another limitation is that data on socio-economic status, education levels, and other demographic factors are self-reported. This can introduce social desirability bias, where participants might overreport favorable behaviours or underreport unfavourable ones.

## 5 Conclusion

The study provides important evidence on the patterns and contextual drivers of child drowning in the remote rural setting of Sandwip Island, Bangladesh. The findings demonstrate that young children, particularly those aged 1–4 years, remain highly vulnerable to drowning, with incidents occurring predominantly in nearby unfenced water bodies during periods of limited adult supervision. The study further highlights how environmental conditions, household routines, and the proximity of ponds to homes collectively increase drowning risk in rural communities.

These findings reinforce the need for integrated child drowning prevention strategies that combine improved supervision, community awareness, safer play environments, and locally appropriate environmental risk reduction measures. Given the high proportion of incidents occurring in unfenced ponds near households, community-based interventions and stronger engagement from local government authorities may play an important role in reducing child drowning risks in geographically isolated rural areas. Overall, the study contributes context-specific evidence that can support the development of targeted drowning prevention policies and interventions in coastal Bangladesh and similar low-resource settings. Children learn to swim at an average age of seven on the island, though they can start at earlier ages, depending on their health (34). Encouraging early swimming skills could reduce drowning incidents. The results highlight the urgent need for drowning prevention experts and practitioners in Bangladesh to take swift action by fostering multi-sectoral collaboration, increasing public awareness, and urging the government to extend the reach of the integrated community-based centers for childcare, protection, and swim-safe facilities to cover additional districts and sub-districts. At the sub-district level, child drowning prevention measures should be incorporated as a regular agenda item in field health offices, teachers’ meetings, and local union council activities.

## Data Availability

The original contributions presented in the study are included in the article/supplementary material, further inquiries can be directed to the corresponding author.
